# Different patterns of introgression in a three species hybrid zone among European cave salamanders

**DOI:** 10.1002/ece3.10437

**Published:** 2023-08-23

**Authors:** Giacomo Bruni, Andrea Chiocchio, Giuseppe Nascetti, Roberta Cimmaruta

**Affiliations:** ^1^ Viale Palmiro Togliatti Sesto Fiorentino Italy; ^2^ Department of Ecological and Biological Sciences Tuscia University Viterbo Italy

**Keywords:** asymmetric introgression, European plethodontids, Italian peninsula, multispecies hybridization, *Speleomantes*

## Abstract

Hybrid zones occur where genetically distinct populations meet, mate and produce offspring with mixed ancestry. In Plethodontid salamanders, introgressive hybridization is a common phenomenon, where hybrids backcross with parental populations leading to the spread of new alleles into the parental genomes. Whereas many hybrid zones have been reported in American Plethodontid salamanders, only a single hybrid zone has been documented in European plethodontids so far, which is located at the Apuan Alps in the Italian Peninsula. Here, we describe a previously unreported hybrid zone in the Northern Apennines involving all the three Plethodontid salamander species inhabiting the Italian Peninsula. We found 21 new *Speleomantes* sites of occurrence, from a hitherto unexplored area located at the boundaries between three *Speleomantes* species ranges. Using mitochondrial (Cytb and ND2 genes) and nuclear markers (two diagnostic SNPs at the NCX1 gene), we revealed a three‐way contact zone where all the three mainland species hybridize: *S. strinatii*, *S. ambrosii* and *S. italicus*. We observed a strong mitonuclear discordance, with mitochondrial markers showing a conspicuous geographic pattern, while diagnostic nuclear SNPs coexisted in both the same populations and individuals, providing evidence of hybridization in many possible combinations. The introgression is asymmetric, with *S. italicus* mitogenome usually associated with *S. a. ambrosii* and, to a lesser extent, to *S. strinatii* nuclear alleles. This finding confirms that Plethodontid are a group of choice to investigate hybridization mechanisms and suggests that behavioural, genetic and ecological components may concur in determining the direction and extent of introgression.

## Graphical abstract

A new hybrid zone in involving all the three Plethodontid salamander species inhabiting the Italian Peninsula is described. We found strong mitonuclear discordance, with mitochondrial markers showing a conspicuous geographic pattern, while discriminant nuclear markers from different species coexisted in both the same populations and individuals, providing evidence of past hybridization in many possible combinations. The introgression pattern is asymmetric, with *S. italicus* mitogenome very often associated with *S*. *a*. *ambrosii* and, to a lesser extent, to *S*. *strinatii* nuclear alleles.

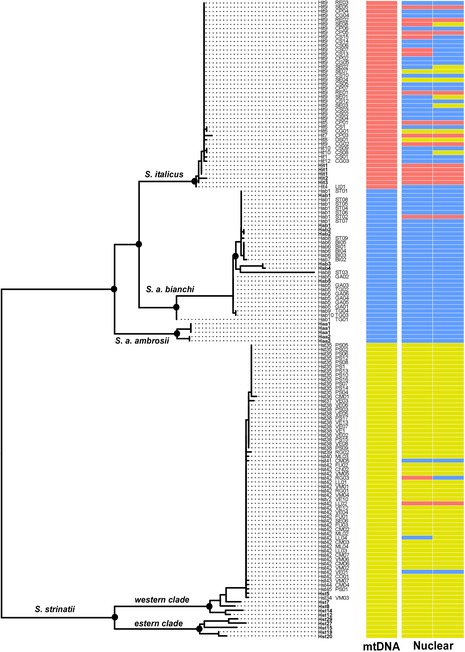

## 1 INTRODUCTION

Hybrid zones are geographic areas where genetically distinct populations meet, mate and produce offspring with mixed ancestry (Barton & Hewitt, 1985). Their study is acknowledged as relevant to understand a wide range of evolutionary mechanisms, such as speciation, reinforcement and adaptation (Hewitt, 2011). At the beginning, hybridization was perceived as a rare event, especially in animals (Harrison, 1993; Mayr, 1963). However, the increasing number of hybrid zones reported in virtually all taxonomic groups promoted hybridization as a widespread phenomenon, occurring at time‐scales from geological to contemporary, and sometimes even between highly differentiated lineages (Harrison & Larson, 2014; Larson et al., 2014; Nosil, 2008; Pyron et al., 2022; Schield et al., 2019). Furthermore, introgressive hybridization, occurring when hybrids backcross with parental populations leading to the spread of foreign alleles into the parental genomes, has been pointed out as a powerful and widespread force introducing adaptive genetic novelties and promoting evolutionary radiations (Barton, 2020; Fraïsse et al., 2014; Seehausen et al., 2014).

Amphibians have provided important insights into hybrid zones research, with cases of stable and moving hybrid zones, and evidence for species replacement and past introgressive hybridization in currently geographically separated taxa (e.g. Arntzen, 1978; Wielstra, Burke, Butlin & Arntzen, 2017, Wielstra, Burke, Butlin, Avcı et al., 2017; Zieliński et al., 2013). Plethodontid salamanders are involved in a relevant number of case studies, fostering the view of hybridization and introgression as evolutionary forces able to increase diversification and speciation rates (Patton et al., 2020). Indeed, plethodontids are both the most speciose and the most prone to hybridization among all the salamander families, although this feature is unevenly distributed among the different lineages (Blankers et al., 2012; Wake, 2009). The high number of hybrid zones observed in plethodontids is largely a result of their low vagility, restricted gene flow and high level of genetic differentiation even across small geographic distances. These features allow climatic/environmental fluctuations to easily isolate populations, triggering their allopatric or parapatric divergence, and then fostering secondary contact and hybridization among diverging lineages without complete reproductive isolation (Vieites et al., 2007). This process of repeated range shifts and secondary contacts under paleoclimatic changes has been inferred in plethodontids from both the American and European continent, witnessing that climate change is one of the strongest drivers of diversification and speciation in this group, and explaining plethodontids' uncommon rate of hybridization (Wiens et al., 2006). Recent studies suggested that 30% of Plethodontid species may hybridize and demonstrated that hybridizing lineages show a higher diversification level, thus comparing the evolutionary role‐played by hybridization in these salamanders similar to that played in haplochromine cichlids (Patton et al., 2020). Accordingly, many iconic case studies showing the relationships between hybridization and diversification of lineages involved Plethodontid salamanders, such as the *Ensatina* ring species, *Plethodon glutinosus* group and the genus *Desmognatus* (Pereira & Wake, 2009; Pyron et al., 2022; Wiens et al., 2006). These features make plethodontids a key group to study the adaptive value of hybridization and its role in determining speciation rates (Patton et al., 2020).

**FIGURE 1:**
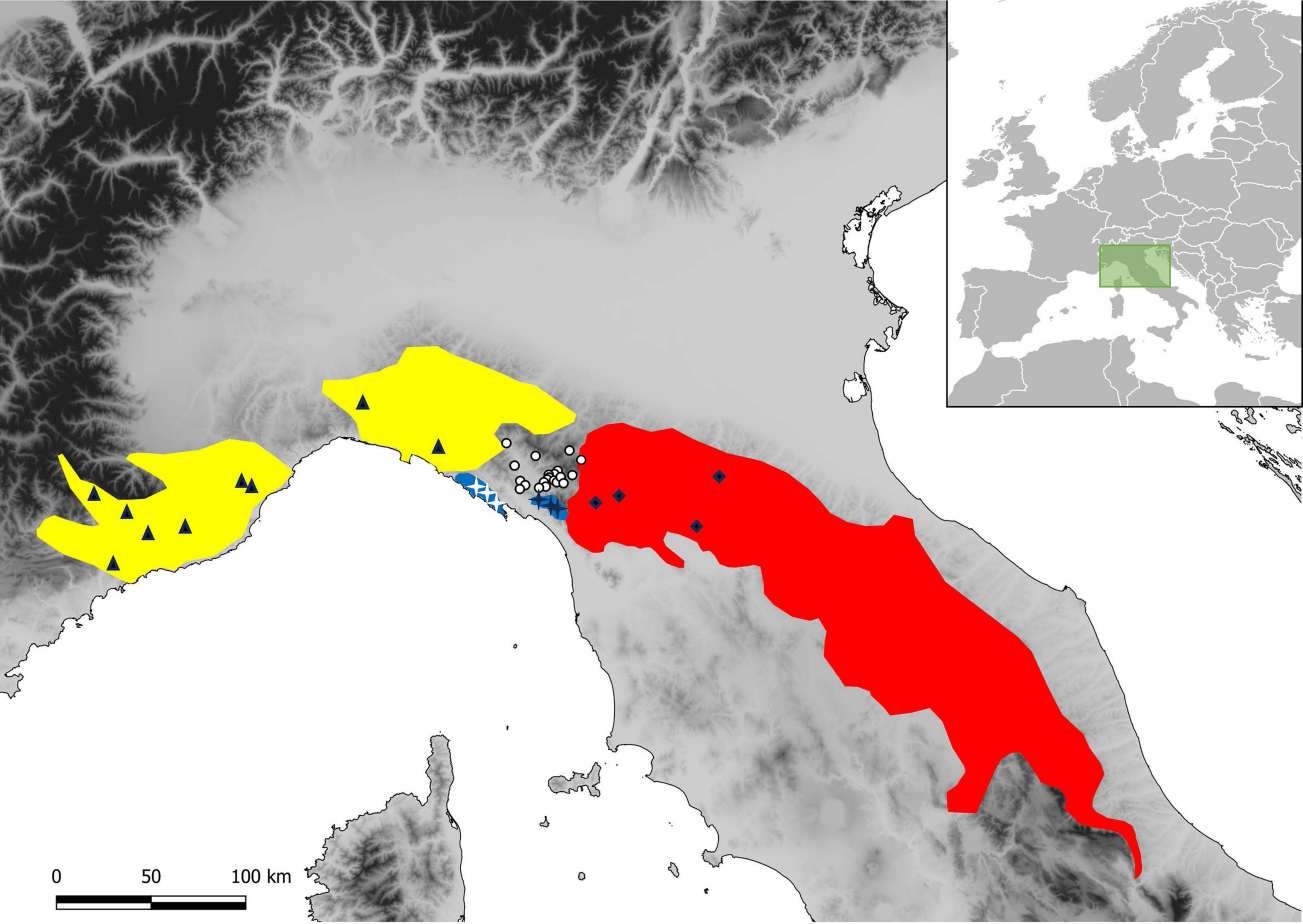
Distribution range of the three mainland species of European cave salamanders: *Speleomantes strinatii* in yellow, *S. ambrosii* in blue, *S. italicus* in red. Sampling sites are shown as white dots. Localities of reference specimens are shown for *S. strinatii* (black triangles), *S. a. ambrosii* (white stars), *S. a. bianchii* (black stars), *S. italicus* (black diamonds).

European *Speleomantes* are the only Plethodontid salamanders present in Europe, accounting for eight species, five of which are endemic to Sardinia. As to the mainland species, *Speleomantes strinatii* (Aellen, 1958) ranges from the northern Apennine to the south‐west of France, while the other two species are endemic to the Apennine Peninsula, namely *S. italicus* (Dunn, 1923) and *S. ambrosii* (Lanza, 1955), this latter with two subspecies, *S. a. ambrosii* and *S. a. bianchii* Lanza et al. (2005) (Figure 1).

*Speleomantes* share the same evolutionary peculiarities of their American relatives, such as small populations and high genetic variation over short geographic distances (Cimmaruta et al., 2015; Pyron et al., 2022). Despite the expectations of common patterns of secondary contact and hybridization in European plethodontids, such as those found in the American plethodontids, only two secondary contact zones have been described so far, both located in peninsular Italy (Cimmaruta et al., 1999; Ruggi et al., 2005). In eastern Liguria, *S. strinatii* and *S. a. ambrosii* are parapatric with alternative fixed alleles and no sign of hybridization, due to interspecific spatial competition (Cimmaruta et al., 1999). Conversely, in the Apuan Alps, *S. a. bianchii* and *S. italicus* hybridize and show a significant asymmetric introgression, with *S. a. bianchii* alleles entering in the gene pool of *S. italicus* for more than 90 km from the contact zone, while a few *S. italicus* alleles hardly spread up to 15 km in the range of *S. a. bianchii* (Ficetola et al., 2019; Forti et al., 1998; Ruggi et al., 2005).

In this study, we tested for the possibility that the three European plethodontid salamanders may interbreed in a secondary contact zone. To this aim, we investigated an area located at the boundaries between the ranges of these species along the Northern Apennines, in central Italy. Using mitochondrial and nuclear markers, we revealed a multiple contact zone, showing that all the three mainland *Speleomantes* species can hybridize and that differential introgressive hybridization occurs, describing uncommon biogeographic and evolutionary patterns in a taxonomic group of interest to evolutionary biologists.

## 2 MATERIALS AND METHODS

### 2.1 Field survey

The field survey was mainly conducted in Tuscany, in an area spanning from the middle‐upper Magra basin, in the Province of Massa‐Carrara, to the extreme north‐western part of the Province of Lucca, where no records of cave salamanders were reported so far (Figure 1; Ambrogio & Mezzadri, 2017; Lanza et al., 2006; Vanni & Nistri, 2006). Additionally, we investigated the area of Collagna (Ventasso, Reggio Emilia) in Emilia‐Romagna, where unidentified cave salamanders were previously reported (Gigante, 2015). Potential suitable sites of occurrence were mostly identified using Google Street View (as in Bruni et al., 2016). The search for *Speleomantes* individuals was carried out in the epigean environment mainly during humid nights by means of headlamp. Cave salamanders leave their underground retreats under moist and cold (but not severe) weather to feed on epigean environment (Lanza et al., 2006). Cave salamanders were captured manually using single‐use nitrile gloves, and tissue samples (a small piece of the tail tip) were collected for further molecular analyses. After the tissue collection procedure, all individuals were released in the same location where they were found. A total of 122 specimens were sampled, and tissues were stored in 96% ethanol until DNA extraction. All the procedures were approved by the Italian Ministry of the Environment MATTM (permits number PNM.0042634 of 07/08/2013, PNM.0006560 of 29/3/2018 and MATTM.0027613 of 16/3/2021), and by the Tuscan‐Emilian Apennine National Park (Prot.N.0001960 of 20/8/2021).

### 2.2 Molecular analyses

Species identification used a molecular‐based approach employing both mitochondrial and nuclear DNA markers.

DNA extractions were performed following the standard cetyltrimethylammonium‐bromide (CTAB) protocol described in Doyle and Doyle (1987). Two mitochondrial DNA (mtDNA) and one nuclear DNA (nDNA) gene fragments were amplified through polymerase chain reaction (PCR) and sequenced using the standard Sanger method. The mtDNA fragments were a 637 bp portion from cytochrome‐b gene (cytb) and a 673 bp portion from the NADH‐dehydrogenase gene, subunit 2 (ND2); PCR conditions followed the protocols described in Cimmaruta et al. (2015). The nDNA fragment was a 525 bp portion from the sodium‐calcium exchanger gene (NCX1); PCR conditions followed the protocols described in Lunghi et al. (2022).

Electropherograms of sequences were visually checked using FinchTV 1.4.0 (Geospiza Inc.) and aligned using Clustal X 2.0 (Larkin et al., 2007). The variation at the nucleotide sequences was assessed using MEGA 6.0 (Tamura et al., 2013); haplotype and nucleotide diversity were estimated using DnaSP 5.10 (Librado & Rozas, 2009). Heterozygous nuclear sequences were phased using the PHASE method (Flot et al., 2006) in DnaSP 5.10 using default parameter values. For each nuclear gene, the probability of recombination was evaluated using the pairwise homoplasy index (PHI statistics; Bruen et al., 2006) in SplitsTree 4.13.1 (Huson & Bryant, 2006). All the subsequent analyses were conducted using phased nuclear data.

We used 27 reference samples of known identity from previous studies to assign both the mitochondrial haplotypes and nuclear alleles found in the 122 samples collected in this study. The reference samples were selected from localities representing the whole range of each species, including those as close as possible to the study area, according to range gaps (Figure 1). In detail, 10 specimens of *S. strinatii* were recovered from Cimmaruta et al. (2015), belonging to both the clades recognised in this species. Five specimens of *S. italicus* were from Lucente et al. (2016) and selected to avoid introgressed genotypes with *S. ambrosii* (Ruggi et al., 2005). Five specimens of *S. ambrosii ambrosii* and seven specimens of *S. ambrosi bianchii* were from sampling zones in the range of a few tens of kilometres from the study area. The five specimens of *S. ambrosii ambrosii* were from Lucente et al. (2016) while the seven specimens of *S. ambrosi bianchii* were previously assigned using allozyme data (Nascetti et al., 1996) and their mt sequences have been obtained for this study and deposited in GenBank (Appendix S1). We estimated the phylogenetic relationships between the 122 new samples and the 27 reference samples using mitochondrial haplotypes under the maximum likelihood (ML) method implemented in IQTREE (Nguyen et al., 2015). The ML tree was inferred setting the best fit model of substitution selected by the MODELFINDER algorithm implemented in IQTREE (−m TEST option); the robustness of the inferred tree topology was assessed using the nonparametric bootstrap method with 1000 pseudoreplicates, and the SH‐like approximate likelihood ratio test (SH‐aLRT), also with 1000 bootstrap replicates. We did not include any outgroup in the analysis.

To test for the presence of hybrids, we employed two diagnostic SNPs (i.e. positions 3 and 192) from the fragment of the nuclear gene NCX1 as described in Lunghi et al. (2022). Briefly, the two SNP positions showed the following pattern in the three Italian *Speleomantes* species: T and C, respectively, in *S. strinatii*, T and T in *S. ambrosii*, C and T in *S. italicus*. We therefore considered as hybrids the specimens resulting heterozygous at the nuclear diagnostic SNPs s and/or homozygous at nuclear SNPs, but showing mtDNA from a distinct species.

## 3 RESULTS

We identified 21 new, previously unreported, cave salamanders' localities of occurrence (Table 1 and Figure 1). The collection points were located between 180 and 970 m asl, and consisted mainly in epigeal environments where the individuals were found both on vertical surfaces (e.g. rocky outcrops and drystone walls) and on the ground (mainly on leaf litter). We collected biological tissue from 122 cave salamanders. From all the individuals, we successfully amplified and sequenced a 637 bp portion from the cytb gene and a 673 bp portion from the ND2 gene; no indels, stop codons or non‐sense codons were observed on either gene. After concatenating the sequences from the two mtDNA genes, we found 28 unique haplotypes, defined by 153 variable positions, 140 of which were parsimony informative. The haplotype diversity (h) and nucleotide diversity (π) values for this data set were 0.870 and 0.048, respectively. The distribution of the haplotypes scored in the sampled localities is shown in Table 1; the GenBank accession numbers are listed in the Appendix S1.

**TABLE 1:** Geographical location of the 21 *Speleomantes* sampling localities investigated in this study, with their sample size and mtDNA haplotype codes.

Site	Acronym	Locality	Latitude	Longitude	N	mtDNA haplotypes
1	CN	Canà	44.39	9.85	1	Hst42
2	ML	Mulazzo‐Lusuolo	44.29	9.91	3	Hst42, Hst40
3	CM	Canala‐Montale	44.22	9.94	7	Hst36, Hst41, Hst42, Hst44
4	CO	Compione	44.33	10.05	1	Hst42
5	VM	Vendaso‐Mommio	44.26	10.19	7	Hst42, Hst34, Hst43
6	FU	La Funicolare	44.24	10.17	3	Hst42
7	VE	Fivizzano‐Verrucola	44.24	10.13	12	Hst37, Hst38, Hst42
8	SE	Serrarola	44.23	10.14	8	Hit9, Hst42
9	CS	Caugliano‐Spicciano	44.22	10.12	15	Hit6, Hit9, Hit10, Hit11
10	GA	Gassano	44.21	10.1	6	Hab5
11	RE	Reusa	44.21	10.17	3	Hit9
12	CP	Casola – Pugliano	44.21	10.19	7	Hit5, Hit7, Hit9
13	RG	Regnano	44.23	10.21	3	Hst39, Hst42
14	CG	Casone Carpinelli – Giuncugnano	44.21	10.23	5	Hit6, Hit9, Hit12
15	DS	Dalli Sotto	44.24	10.29	1	Hit8
16	TG	La Traggiara – La Giunca	44.19	10.11	4	Hab5, Hab10, Hab11
17	ST	Bardine di San Terenzo	44.18	10.07	9	Hab1, Hab8
18	BI	Bibola	44.19	9.97	5	Hab6, Hab7
19	PS	Caprigliola – Ponzano Superiore	44.17	9.94	17	Hst35, Hst38, Hst45
20	LL	Collagna	44.36	10.27	4	Hst42
21	LI	Ligonchio	44.31	10.35	1	Hit4

From all the individuals, we successfully amplified and sequenced a 525 bp portion of the NCX1 nuclear gene. After the phasing procedure, we retrieved 27 unique haplotypes defined by 38 variable positions. All the haplotypes showed the SNP diagnostic combinations at positions 3 and 192 as described in Lunghi et al. (2022).

**FIGURE 2:**
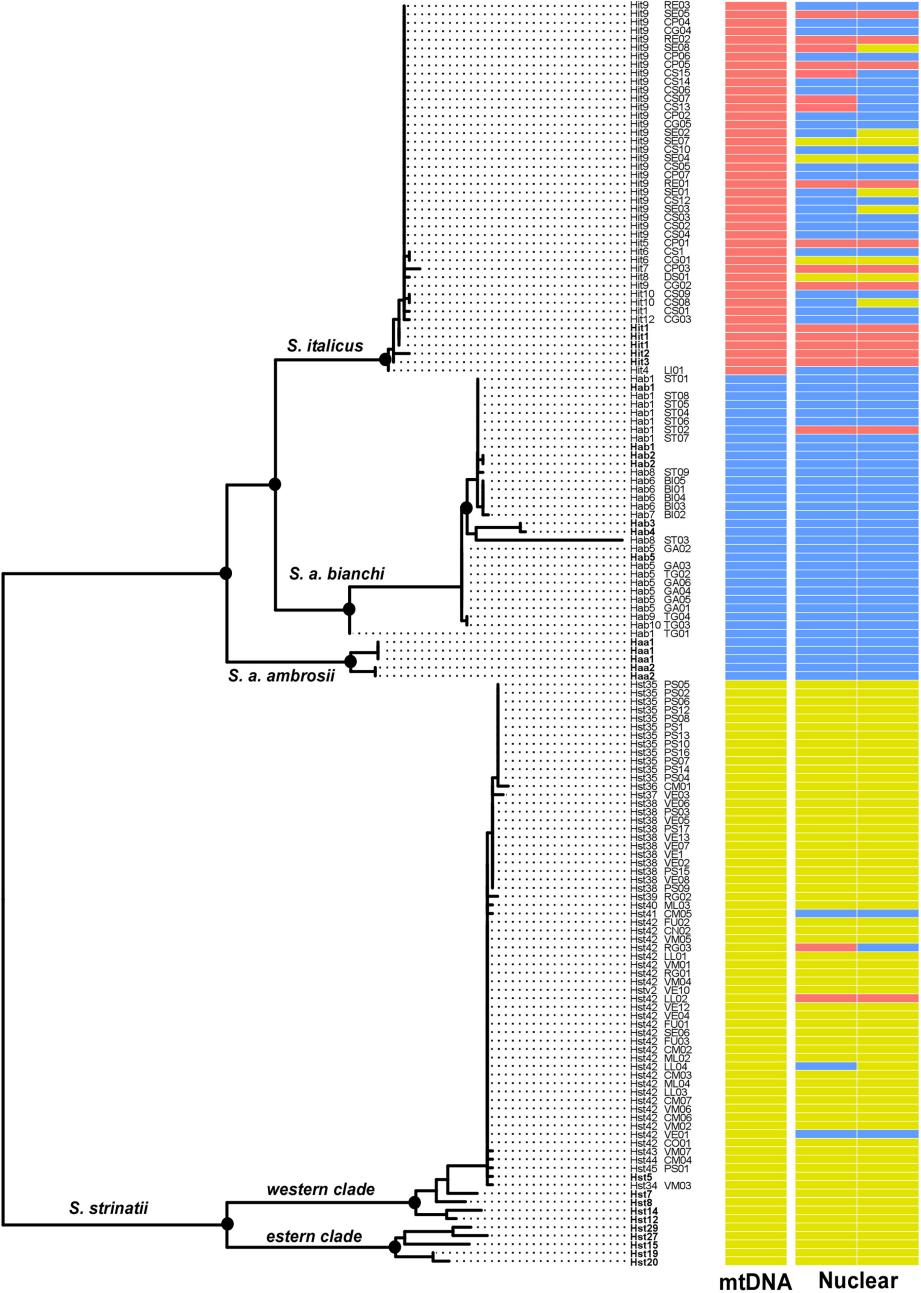
Maximum Likelihood tree obtained from IQTREE and showing the phylogenetic relationships among mtDNA haplotypes from the reference individuals (in bold) and from the 122 cave salamanders found in the study area; dotted nodes indicate >95% support at both SH‐aLRT and nonparametric bootstrap support over 1000 replicates. The bar‐plot on the right indicates the individual genotype at mtDNA and nuclear (NCX1) markers, using the same colours as in Figure 1.

The ML tree of the mtDNA sequences obtained by IQTREE (log‐likelihood score: −3508.067; s.e. 120.097) clearly defined four distinct, fully supported main branches, corresponding to the four Italian cave salamander taxa *S. strinatii*, *S. ambrosii ambrosii*, *S. ambrosii bianchi* and *S. italicus* (Figure 2). The *S. strinatii* branch showed a deep split in two distinct haplogroups, corresponding to the eastern and western clades of Cimmaruta et al. (2015). *Speleomantes ambrosii* and *S. italicus* clustered together, with the phylogenetic relationships among *S. a. ambrosii*, *S. a. bianchi* and *S. italicus* not fully resolved. Indeed, *S. a. ambrosii* resulted sister of a clade made of *S. a. bianchi* + *S. italicus*, in line with the results obtained by Carranza et al. (2008) with a different set of genes. No substantial differentiation has been found within *S. italicus*.

**FIGURE 3:**
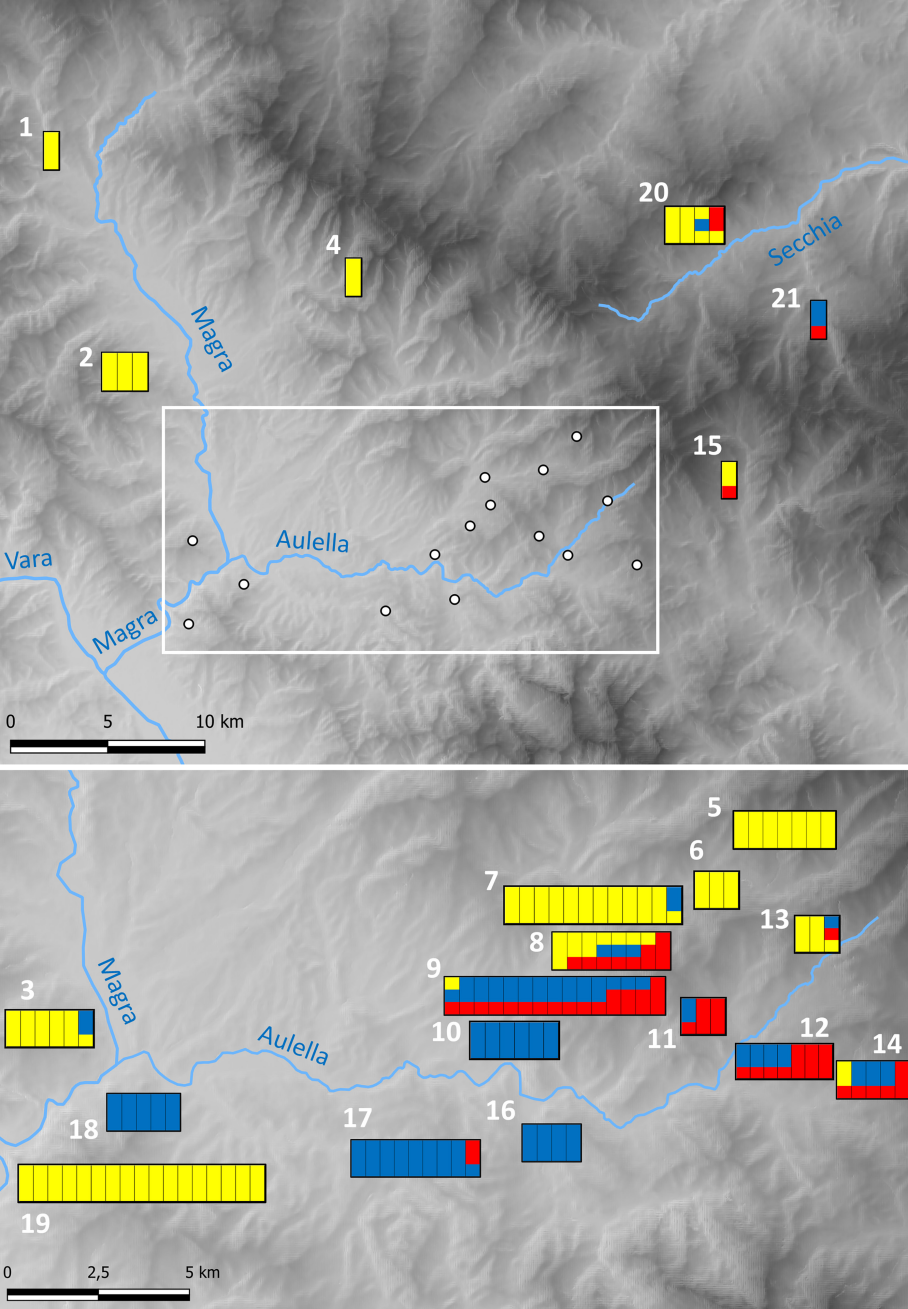
Multilocus genotypes of the studied salamanders. Each bar represents a specimen localized in its sampling locality, numbered as in Table 1. Each bar indicates the individual genotype at mitochondrial marker (bottom) and at the two discriminant SNPs at the nuclear marker NCX1, using the same colours as in Figure 1. The main watercourses are highlighted and named.

The mtDNA haplotypes from the 122 individuals of unknown identity clustered with *S. italicus*, *S. a. bianchii* and *S. strinatii* eastern clade, unveiling the presence of a tight zone of sympatry among the three species in less than 50 km: 39 individuals clustered with *S. italicus* (mean genetic distance 0.001, sd 0.000), 24 individuals clustered with *S. a. bianchii* (mean genetic distance 0.014, sd 0.002) and 59 individuals clustered with *S. strinatii* eastern clade (mean genetic distance 0.009, sd 0.001). In the northern and westernmost localities (1–7, 13, 19, 20), we found *S. strinatii* haplotypes; in the southwestern localities (9, 11, 12, 14, 15, 21), we found *S. italicus* haplotypes, whereas *S. a. bianchii* haplotypes have been found only in four sites (10, 16–18). Both *S. strinatii* and *S. italicus* haplotypes coexisted only at site 8 (Figure 3).

Results from the sequencing of the NCX1 nuclear gene identified a hybrid zone among the three cave salamander species in the study area, revealing the presence of a complex pattern of hybridization and introgression. Among the individuals with *S. strinatii* mtDNA, three showed *S. ambrosii* nuclear haplotypes, one showed *S. italicus* nuclear haplotypes and another one showed both *S. italicus* and *S. ambrosii* nuclear haplotypes. A single individual with *S. ambrosii* mtDNA haplotype showed *S. italicus* nuclear haplotypes. Thirty‐two out of 39 individuals with *S. italicus* mtDNA haplotypes showed *S. ambrosii* or *S. strinatii* nuclear haplotypes, four of which had both *S. ambrosii* and *S. strinatii* nuclear haplotypes. Accordingly, most of individuals from the localities 8, 9, 12, 13, 14 and 20 had admixed genotype among two or three species, representing one of the most complex hybrid zones in the Italian peninsula (Figure 3b).

## 4 DISCUSSION

Our molecular analysis allowed identifying a new hybrid zone between the three mainland *Speleomantes* salamanders, a multispecies pattern never observed before in European plethodontids. The introgression pattern observed revealed the presence of a strong mitonuclear discordance. Mitochondrial markers were subdivided, with sampling localities largely characterized by the presence of haplotypes belonging to a single species. On the contrary, diagnostic nuclear SNPs coexisted in both the same populations and individuals (Figure 3). Hybridization events involving multiple species have not previously been observed in European plethodontid salamanders while already described in many American plethodontids. For example, the discordance due to the presence of mtDNA haplotypes from four divergent clades in *Plethodon shermani* coupled with population homogeneity evidenced by allozymes was explained by introgressive hybridization events with *P. jordani* and numerous species of the *P. glutinosus* complex (Weisrock et al., 2005). The discordance among mitochondrial vs. nuclear and colour‐pattern traits detected in a three‐species hybrid zone among *Plethodon jordani*, *P. metcalfi* and *P. teyahalee* from the southern Appalachian Mountains was also explained by differential introgression after repeated cycles of isolation and secondary contacts producing a mosaic pattern of hybridization (Chatfield et al., 2010). Recent studies are increasingly detecting the signature of multiple hybridization events in plethodontids, as in the case of the contact zones occurring among the 47 putative species belonging to the genus *Desmognathus*, showing that hybridization occurred in at least six distinct lineages, with some evidence of high admixture levels involving four to five species (Pyron et al., 2022).

Our data show that the three *Speleomantes* species lack effective prezygotic reproductive barriers because signatures of hybridization are found in all their gene pools in many possible combinations. Nonetheless, the introgression is neither free nor symmetric, with *S. italicus* mitogenome very often associated with S. *a. ambrosii* and, to a lesser extent, to *S. strinatii* nuclear alleles, while the opposite rarely occurred. Over the 39 specimens having *S. italicus* mitochondrial haplotypes, only seven were homozygous for nuclear *italicus* alleles. Conversely, a single specimen of *S. a. ambrosii* (out of 24) and five *S. strinatii* (out of 59) showed introgressed genotypes. The only other hybrid zone described so far in European plethodontids, located on the Apuan Alps, showed mitonuclear discordance and asymmetric introgression as well. The pattern observed at nuclear allozyme markers evidenced *S. a. bianchii* alleles strongly entering *S. italicus* gene pool and moving tens of kilometres away from the contact zone, while mtDNA showed a clean‐cut subdivision with nearby populations having alternatively *S. a. bianchii* or *S. italicus* haplotyes (Nascetti et al., 1996; Ruggi et al., 2005). A similar pattern was evidenced also by an experiment of artificial syntopy between *S. italicus* and *S. a. ambrosii* in a cave located outside *Speleomantes* range and carried on for 30 years (Cimmaruta et al., 2013; Forti et al., 2005). The results showed that the two taxa were able to produce viable hybrids and recombinants, but also that the crosses have been asymmetric, with pure *S. a. ambrosii* constituting the bulk of the population and the recombinant genotypes largely characterized by *S. ambrosii* nuclear alleles associated with *S. italicus* mitochondrial haplotypes (Cimmaruta et al., 2013). These findings provide support for a lack of reproductive barriers in these species but that, at the same time, the gene exchange between these taxa is restricted since alleles from distinct lineages coexist mainly within *S. italicus* gene pool.

Many possible mechanisms, likely co‐occorring, could explain the observed pattern of introgression (Canestrelli et al., 2016; Canestrelli et al., 2017; Petit & Excoffier, 2009; Toews & Brelsford, 2012). Sex biased crosses may be partially responsible: if *S. italicus* females would be more receptive towards heterospecific crosses, this would explain the massive coexistence of *S. italicus* mtDNA with *ambrosii/strinatii* nuclear alleles. In support of this idea, it worths noting that in experiments of artificial syntopy between *S. italicus* and *S. a. ambrosii* the first crosses occurred between *S. italicus* females x *S. a. ambrosii* males (Cimmaruta et al., 2013; Forti et al., 2005). Furthermore, a possible unbalance among the densities of the different species may induce *S. italicus* females to accept heterospecific mates if they constitute the most frequent encounters. The capture of genetic variation following secondary contacts and subsequent introgressive hybridization has been commonly observed in salamanders and postulated as a relevant force in shaping their extant geographic pattern of genetic diversity (Chiocchio et al., 2021, 2022; Patton et al., 2020). *S. italicus* is characterized by a very low genetic diversity when compared with the other mainland species, as evidenced by different markers, from allozymes to mitochondrial RFLPs to sequences of both nuclear and mitochondrial genes (Lanza et al., 2005; Nascetti et al., 1996; Ruggi et al., 2005). Therefore, introgression may result in an increase of genetic diversity in *S. italicus*, especially in front of a low genomic coadaptation. This is also the case of *S. strinatii*, whose westernmost lineages showed admixture coupled with a high genetic variation that was explained by multiple secondary contacts among subclades, opposite to the low genetic variation of the easternmost lineage that did not show evidence of secondary contacts (Cimmaruta et al., 2015). The data presented here are too preliminary to test for this hypothesis, since only three loci (two mitochondrial and one nuclear) have been used, thus preventing further analyses. However, the adaptive nature of introgressive hybridization has been documented in plethodontids and has been shown to facilitate diversification, overall promoting speciation and decreasing extinction rates (Patton et al., 2020). In some cases, it has been demonstrated that hybrids have a higher fitness than the parental genotypes, as in the hybrid zone between *Plethodon shermani* and *P. teyahalee* along an altitudinal gradient. Phenotypic hybrids showed higher survival rates at low elevation with respect to parental *P. shermani* since *P. teyahalee* is better adapted to warmer and dryer environments (Gade et al., 2022). The possibility that introgressive hybridization may confer an advantage to the studied *Speleomantes* species has been recently supported by studying niche width in parental and introgressed populations of *S. italicus* and *S. a. bianchii* from the contact zone located on the Apuan Alps (Ficetola et al., 2019). The distribution of the specimens along the gradient of humidity, temperature and incident light recorded within the caves showed that hybrids were able to distribute in drier, warmer, and more luminous sectors with respect to the parental species, so exhibiting a transgressive niche expanded towards harsher environmental conditions (Ficetola et al., 2019). These data suggest that introgressive hybridization may favour hybrids both presently, by allowing the exploitation of less favourable habitats (cave entrance) where, however, the density of prey is more abundant, and in future, since the enhanced tolerance to harsh conditions may constitute a pre‐adaptation to future climatic scenarios.

As a final remark, our work allowed updating the ranges of *S. italicus*, *S. ambrosii* and *S. strinatii*, by identifying 21 populations in a zone where no *Speleomantes* were previously known. This finding significantly extends the known distribution for both *S. a. bianchii* and *S. strinatii* and contribute to the implementation of the new Atlas of Amphibians and Reptiles of Italy (Sindaco et al., 2022). The proper identification of species boundaries may be particularly relevant for conservation purposes in the face of the ongoing widespread decline of amphibians.

## 5 CONCLUSIONS

The pattern of introgression observed in the hybrid zone between *S. italicus*, *S. a. bianchii* and *S. strinatii* here studied is concordant with previous studies suggesting that hybrid populations may themselves constitute a filter to gene flow, able to prevent the free merging of the involved gene pools (Barth et al., 2020; Martinsen et al., 2001). At the same time, indirect evidence drawn from case studies regarding American plethodontids, and a comparison with the only other hybrid zone so far described among European plethodontids, both suggest that behavioural, genetic and ecological mechanisms are involved in determining the direction and extent of introgression, including sex biased crosses, endogenous factors and adaptive hybridization with possible niche transgression. Further investigations involving phenotypic characterization coupled with genome‐scale sequencing approaches could provide new insights into the genomic architecture of this hybrid zone formation, as well as on the adaptive values of introgressive hybridization in European plethodontids.

## AUTHOR CONTRIBUTIONS

**Giacomo Bruni:** Conceptualization (equal); data curation (lead); formal analysis (supporting); investigation (equal); methodology (equal); software (supporting); visualization (equal); writing – original draft (equal); writing – review and editing (equal). **Andrea Chiocchio:** Conceptualization (equal); data curation (equal); formal analysis (lead); investigation (equal); methodology (equal); software (equal); validation (equal); visualization (equal); writing – original draft (equal); writing – review and editing (equal). **Giuseppe Nascetti:** Conceptualization (lead); funding acquisition (equal); investigation (equal); supervision (equal); writing – review and editing (equal). **Roberta Cimmaruta:** Conceptualization (lead); formal analysis (equal); funding acquisition (lead); investigation (equal); project administration (lead); resources (lead); validation (lead); writing – original draft (lead); writing – review and editing (equal).

## CONFLICT OF INTEREST STATEMENT

The authors declare no competing interests.

## Supporting information


Appendix S1.

## Data Availability

All genetic sequences were submitted to GenBank. The accession numbers of gene sequences are listed in the Appendix S1.

## References

[ece310437-bib-0001] Ambrogio, A. , & Mezzadri, S. (2017). Geotritoni d'Italia – Cave salamanders of Italy (p. 64). Gavia Edizioni.

[ece310437-bib-0002] Arntzen, J. W. (1978). Some hypotheses on postglacial migrations of the fire‐bellied toad, *Bombina bombina* (Linnaeus) and the yellow‐bellied toad, *Bombina variegata* (Linnaeus). Journal of Biogeography, 5, 339–345.

[ece310437-bib-0003] Barth, J. M. I. , Gubili, C. , Matschiner, M. , Tørresen, O. K. , Watanabe, S. , Egger, B. , Han, Y.‐S. , Feunteun, E. , Sommaruga, R. , Jehle, R. , & Schabetsberger, R. (2020). Stable species boundaries despite ten million years of hybridization in tropical eels. Nature Communications, 11, 1433. 10.1038/s41467-020-15099-x PMC7080837 32188850

[ece310437-bib-0004] Barton, N. H. (2020). On the completion of speciation. Philosophical Transactions of the Royal Society B, 375(1806), 20190530. 10.1098/rstb.2019.0530 PMC7423282 32654647

[ece310437-bib-0005] Barton, N. H. , & Hewitt, G. M. (1985). Analysis of hybrid zones. Annual Review of Ecology and Systematics, 16(1), 113–148.

[ece310437-bib-0006] Blankers, T. , Adams, D. C. , & Wiens, J. J. (2012). Ecological radiation with limited morphological diversification in salamanders. Journal of Evolutionary Biology, 25(4), 634–646. 22268991 10.1111/j.1420-9101.2012.02458.x

[ece310437-bib-0007] Bruen, T. C. , Philippe, H. , & Bryant, D. (2006). A simple and robust statistical test for detecting the presence of recombination. Genetics, 172, 2665–2681. 16489234 10.1534/genetics.105.048975 PMC1456386

[ece310437-bib-0008] Bruni, G. , Novaga, R. , Fiacchini, D. , Spilinga, C. , & Domeneghetti, D. (2016). Updated distribution of *Hydromantes italicus* Dunn, 1923 (Caudata Plethodontidae): A review with new records and the first report for Latium (Italy). Biodiversity Journal, 7, 347–352.

[ece310437-bib-0009] Canestrelli, D. , Bisconti, R. , Chiocchio, A. , Maiorano, L. , Zampiglia, M. , & Nascetti, G. (2017). Climate change promotes hybridisation between deeply divergent species. PeerJ, 5, e3072. 28348926 10.7717/peerj.3072 PMC5366042

[ece310437-bib-0010] Canestrelli, D. , Porretta, D. , Lowe, W. H. , Bisconti, R. , Carere, C. , & Nascetti, G. (2016). The tangled evolutionary legacies of range expansion and hybridization. Trends in Ecology & Evolution, 31(9), 677–688. 27450753 10.1016/j.tree.2016.06.010

[ece310437-bib-0011] Carranza, S. , Romano, A. , Arnold, E. N. , & Sotgiu, G. (2008). Biogeography and evolution of European cave salamanders, *Hydromantes* (Urodela: Plethodontidae), inferred from mtDNA sequences. Journal of Biogeography, 35(4), 724–738.

[ece310437-bib-0012] Chatfield, M. W. H. , Kozak, K. H. , Fitzpatrick, B. M. , & Tucker, P. K. (2010). Patterns of differential introgression in a salamander hybrid zone: Inferences from genetic data and ecological niche modelling. Molecular Ecology, 19, 4265–4282. 20819165 10.1111/j.1365-294X.2010.04796.x PMC5478279

[ece310437-bib-0013] Chiocchio, A. , Arntzen, J. W. , Martínez‐Solano, I. , Vries, W. , Bisconti, R. , Pezzarossa, A. , Maiorano, L. , & Canestrelli, D. (2021). Reconstructing hotspots of genetic diversity from glacial refugia and subsequent dispersal in Italian common toads (*Bufo bufo*). Scientific Reports, 11, 260. 10.1038/s41598-020-79046-y 33420098 PMC7794404

[ece310437-bib-0014] Chiocchio, A. , de Rysky, E. , Carere, C. , Nascetti, G. , Bisconti, R. , & Canestrelli, D. (2022). Behavioural foundation of a massive mitochondrial introgression in the fire salamander, *Salamandra Salamandra* . bioRxiv. 10.1101/2022.08.03.502637

[ece310437-bib-0015] Cimmaruta, R. , Forti, G. , Lucente, D. , & Nascetti, G. (2013). Thirty years of artificial syntopy between *Hydromantes italicus* and *H. ambrosii ambrosii* (Amphibia, Plethodontidae). Amphibia‐Reptilia, 34(3), 413–420.

[ece310437-bib-0016] Cimmaruta, R. , Forti, G. , Nascetti, G. , & Bullini, L. (1999). Spatial distribution and competition in two parapatric sibling species of European plethodontid salamanders. Ethology Ecology & Evolution, 11(4), 383–398.

[ece310437-bib-0017] Cimmaruta, R. , Lucente, D. , & Nascetti, G. (2015). Persistence, isolation and diversification of a naturally fragmented species in local refugia: The case of *Hydromantes strinatii* . PLoS One, 10(6), e0131298. 26107249 10.1371/journal.pone.0131298 PMC4479377

[ece310437-bib-0018] Doyle, J. , & Doyle, J. (1987). A rapid DNA isolation procedure for small quantities of fresh leaf tissue. Phytochemical Bulletin, 19, 11–15.

[ece310437-bib-0019] Ficetola, G. F. , Lunghi, E. , Cimmaruta, R. , & Manenti, R. (2019). Transgressive niche across a salamander hybrid zone revealed by microhabitat analyses. Journal of Biogeography, 46(7), 1342–1354.

[ece310437-bib-0020] Flot, J. F. , Tillier, A. , Samadi, S. , & Tillier, S. (2006). Phase determination from direct sequencing of length‐variable DNA regions. Molecular Ecology Notes, 6, 627–630.

[ece310437-bib-0021] Forti, G. , Cimmaruta, R. , Nascetti, G. , Lanza, B. , & Bullini, L. (1998). Glaciazioni del Quaternario e microevoluzione delle popolazioni continentali del genere *Hydromantes* (Amphibia, Plethodontidae). Biogeographia–The Journal of Integrative Biogeography, 19(1), 197–211.

[ece310437-bib-1001] Forti, G. , Lanza, B. , Cimmaruta, R. , & Nascetti, G. (2005). An experiment of artificial syntopy ex situ between Speleomantes italicus and *S. ambrosii ambrosii* (Amphibia, *Plethodontidae*). In G. Doria (Ed.), Annali Museo Civico Storia Naturale G. Doria (Vol. 97, pp. 123–133). The Museum.

[ece310437-bib-0022] Fraïsse, C. , Roux, C. , Welch, J. J. , & Bierne, N. (2014). Gene‐flow in a mosaic hybrid zone: Is local introgression adaptive? Genetics, 197(3), 939–951. 24788603 10.1534/genetics.114.161380 PMC4096372

[ece310437-bib-0023] Gade, M. R. , Zhao, Q. , & Peterman, W. E. (2022). Spatial variation in demographic processes and the potential role of hybridization for the future. Landscape Ecology, 37(10), 2671–2687.

[ece310437-bib-0024] Gigante, M. (2015). Il genere *Hydromantes* (Gistel, 1848) in Emilia‐Romagna – Note su ecologia, conservazione e aggiornamento sulla distribuzione. Speleologia Emiliana, 6(5), 47–62.

[ece310437-bib-0025] Harrison, R. G. (1993). Hybrids and hybrid zones: Historical perspective. Hybrid zones and the evolutionary process (pp. 3–12). Oxford University Press.

[ece310437-bib-0026] Harrison, R. G. , & Larson, E. L. (2014). Hybridization, introgression, and the nature of species boundaries. Journal of Heredity, 105(S1), 795–809. 25149255 10.1093/jhered/esu033

[ece310437-bib-0027] Hewitt, G. M. (2011). Quaternary phylogeography: The roots of hybrid zones. Genetica, 139, 617–638. 21234647 10.1007/s10709-011-9547-3

[ece310437-bib-0028] Huson, D. H. , & Bryant, D. (2006). Application of phylogenetic networks in evolutionary studies. Molecular Biology and Evolution, 23(2), 254–267. 16221896 10.1093/molbev/msj030

[ece310437-bib-1002] Lanza, B. , Cimmaruta, R. , Forti, G. , Bullini, L. , & Nascetti, G. (2005). Bianchi's cave salamander, Speleomantes ambrosii bianchii n. ssp. (Amphibia, *Caudata*, *Plethodontidae*). In G. Doria (Ed.), Annali del Museo Civico di Storia Naturale Giacomo Doria (Vol. 97, pp. 59–77). The Museum.

[ece310437-bib-0029] Lanza, B. , Pastorelli, C. , Laghi, P. , & Cimmaruta, R. (2006). A review of systematics, taxonomy, genetics, biogeography and natural history of the genus *Speleomantes* Dubois, 1984 (Amphibia Caudata Plethodontidae). Atti del Museo Civico di Storia Naturale di Trieste, 52, 5–135.

[ece310437-bib-0030] Larkin, M. A. , Blackshields, G. , Brown, N. P. , Chenna, R. , McGettigan, P. A. , McWilliam, H. , Valentin, F. , Wallace, I. M. , Wilm, A. , Lopez, R. , Thompson, J. D. , Gibson, T. J. , & Higgins, D. G. (2007). Clustal W and Clustal X version 2.0. Bioinformatics, 23(21), 2947–2948. 17846036 10.1093/bioinformatics/btm404

[ece310437-bib-0031] Larson, E. L. , White, T. A. , Ross, C. L. , & Harrison, R. G. (2014). Gene flow and the maintenance of species boundaries. Molecular Ecology, 23(7), 1668–1678. 24795995 10.1111/mec.12601

[ece310437-bib-0032] Librado, P. , & Rozas, J. (2009). DnaSP v5: A software for comprehensive analysis of DNA polymorphism data. Bioinformatics, 25(11), 1451–1452. 19346325 10.1093/bioinformatics/btp187

[ece310437-bib-1003] Lucente, D. , Renet, J. , Gailledrat, M. , Tillet, J. , Nascetti, G. , & Cimmaruta, R. (2016). A new population of European cave salamanders (genus *Hydromantes*) from west‐central France: relict or introduction. The Herpetological Bulletin, 138, 21–23.

[ece310437-bib-0033] Lunghi, E. , Manenti, R. , & Cimmaruta, R. (2022). The identity of an allochthonous Pyrenean population of *Speleomantes* cave salamanders. Salamandra, 58(1), 67–70.

[ece310437-bib-0034] Martinsen, G. D. , Whitham, T. G. , Turek, R. J. , & Keim, P. (2001). Hybrid populations selectively filter gene introgression between species. Evolution, 55(7), 1325–1335. 11525457 10.1111/j.0014-3820.2001.tb00655.x

[ece310437-bib-0035] Mayr, E. (1963). Animal species and evolution. Harvard University Press.

[ece310437-bib-0036] Nascetti, G. , Cimmaruta, R. , Lanza, B. , & Bullini, L. (1996). Molecular taxonomy of European plethodontid salamanders (genus *Hydromantes*). Journal of Herpetology, 30(2), 161–183.

[ece310437-bib-0037] Nguyen, L.‐T. , Schmidt, H. A. , von Haeseler, A. , & Minh, B. Q. (2015). IQ‐TREE: A fast and effective stochastic algorithm for estimating maximum likelihood phylogenies. Molecular Biology and Evolution, 32, 268–274. 10.1093/molbev/msu300 25371430 PMC4271533

[ece310437-bib-1004] Nosil, P. (2008). Speciation with gene flow could be common. Molecular Ecology, 17(9), 2103–2106. 10.1111/j.1365-294X.2008.03715.x 18410295

[ece310437-bib-0038] Patton, A. H. , Margres, M. J. , Epstein, B. , Eastman, J. , Harmon, L. J. , & Storfer, A. (2020). Hybridizing salamanders experience accelerated diversification. Scientific Reports, 10(1), 1–12. 32300150 10.1038/s41598-020-63378-w PMC7162952

[ece310437-bib-0039] Pereira, R. J. , & Wake, D. B. (2009). Genetic leakage after adaptive and nonadaptive divergence in the *Ensatina eschscholtzii* ring species. Evolution, 63(9), 2288–2301. 19453728 10.1111/j.1558-5646.2009.00722.x

[ece310437-bib-0040] Petit, R. J. , & Excoffier, L. (2009). Gene flow and species delimitation. Trends in Ecology & Evolution, 24(7), 386–393. 19409650 10.1016/j.tree.2009.02.011

[ece310437-bib-0041] Pyron, R. A. , O'Connell, K. A. , Lemmon, E. M. , Lemmon, A. R. , & Beamer, D. A. (2022). Candidate‐species delimitation in *Desmognathus* salamanders reveals gene flow across lineage boundaries, confounding phylogenetic estimation and clarifying hybrid zones. Ecology and Evolution, 12(2), e8574. 35222955 10.1002/ece3.8574 PMC8848459

[ece310437-bib-0043] Ruggi, A. , Cimmaruta, R. , Forti, G. , & Nascetti, G. (2005). Preliminary study of a hybrid zone between *Speleomantes italicus* Dunn 1923 and *S. ambrosii* Lanza 1955 on the Apuan Alps, using RLFP analysis. Annali del Museo Civico di Storia Naturale Giacomo Doria Genova, 97, 135–144.

[ece310437-bib-0044] Schield, D. R. , Perry, B. W. , Adams, R. H. , Card, D. C. , Jezkova, T. , Pasquesi, G. I. , Nikolakis, Z. L. , Row, K. , Meik, J. M. , Smith, C. F. , Mackessy, S. P. , & Castoe, T. A. (2019). Allopatric divergence and secondary contact with gene flow: A recurring theme in rattlesnake speciation. Biological Journal of the Linnean Society, 128(1), 149–169.

[ece310437-bib-0045] Seehausen, O. , Butlin, R. K. , Keller, I. , Wagner, C. E. , Boughman, J. W. , Hohenlohe, P. A. , Peichel, C. L. , Saetre, G. P. , Claudia Bank , Brännström, A. , Brelsford, A. , Clarkson, C. S. , Eroukhmanoff, F. , Feder, J. L. , Fischer, M. C. , Foote, A. D. , Franchini, P. , Jiggins, C. D. , Jones, F. C. , … Widmer, A. (2014). Genomics and the origin of species. Nature Reviews Genetics, 15(3), 176–192. 10.1038/nrg3644 24535286

[ece310437-bib-0046] Sindaco, R. , Bruni, G. , Domeneghetti, D. , Liuzzi, C. , Razzetti, E. , Restivo, S. , & Seglie, D. (2022). Il Nuovo Atlante degli Anfibi e dei Rettili d'Italia. Il Naturalista Siciliano, XLVI, 385–390. 10.5281/zenodo.6790636

[ece310437-bib-0048] Tamura, K. , Stecher, G. , Peterson, D. , Filipski, A. , & Kumar, S. (2013). MEGA6: Molecular evolutionary genetics analysis version 6.0. Molecular Biology and Evolution, 30(12), 2725–2729. 24132122 10.1093/molbev/mst197 PMC3840312

[ece310437-bib-0049] Toews, D. P. , & Brelsford, A. (2012). The biogeography of mitochondrial and nuclear discordance in animals. Molecular Ecology, 21, 3907–3930. 22738314 10.1111/j.1365-294X.2012.05664.x

[ece310437-bib-0050] Vanni, S. , & Nistri, A. (2006). Atlante degli anfibi e dei rettili della Toscana. Museo di Storia Naturale dell‟Università degli Studi di Firenze, sezione di Zoologia “La Specola”. Edizioni Regione Toscana.

[ece310437-bib-0051] Vieites, D. R. , Min, M. S. , & Wake, D. B. (2007). Rapid diversification and dispersal during periods of global warming by plethodontid salamanders. Proceedings of the National Academy of Sciences, 104, 19903–19907. 10.1073/pnas.0705056104 PMC2148395 18077422

[ece310437-bib-0052] Wake, D. B. (2009). What salamanders have taught us about evolution. Annual Review of Ecology, Evolution, and Systematics, 40, 333–352.

[ece310437-bib-0053] Weisrock, D. W. , Kozak, K. H. , & Larson, A. (2005). Phylogeographic analysis of mitochondrial gene flow and introgression in the salamander, *Plethodon shermani* . Molecular Ecology, 14, 1457–1472. 15813784 10.1111/j.1365-294X.2005.02524.x

[ece310437-bib-0054] Wielstra, B. , Burke, T. , Butlin, R. K. , & Arntzen, J. W. (2017). A signature of dynamic biogeography: Enclaves indicate past species replacement. Proceedings of the Royal Society of London B: Biological Sciences, 284(1868), 20172014. 10.1098/rspb.2017.2014 PMC5740283 29187631

[ece310437-bib-0055] Wielstra, B. , Burke, T. , Butlin, R. K. , Avcı, A. , Üzüm, N. , Bozkurt, E. , Olgun, K. , & Arntzen, J. W. (2017). A genomic footprint of hybrid zone movement in crested newts. Evolution Letters, 1(2), 93–101. 30283642 10.1002/evl3.9 PMC6121819

[ece310437-bib-0056] Wiens, J. J. , Engstrom, T. N. , & Chippindale, P. T. (2006). Rapid diversifcation, incomplete isolation, and the “speciation clock” in North American salamanders (genus *Plethodon*): Testing the hybrid swarm hypothesis of rapid radiation. Evolution, 60, 2585–2603. 17263119

[ece310437-bib-0057] Zieliński, P. , Nadachowska‐Brzyska, K. , Wielstra, B. , Szkotak, R. , Covaciu‐Marcov, S. , Cogălniceanu, D. , & Babik, W. (2013). No evidence for nuclear introgression despite complete mtDNA replacement in the Carpathian newt (*Lissotriton montandoni*). Molecular Ecology, 22(7), 1884–1903. 23379646 10.1111/mec.12225

