# Supplementary material for: Different patterns of introgression in a three species hybrid zone among European cave salamanders
Source: Ecol Evol. 2023 Aug 23;13(8):e10437. doi: 10.1002/ece3.10437 (PMC10447881; doi:10.1002/ece3.10437)
Supplement: Supplementary file 1 — Appendix S1. [file ECE3-13-e10437-s001.pdf]

Appendix 1 - GENBANK accession numbers for all the DNA sequences of the *Speleomantes* individuals analysed for the present study

Site code	Specimen	MtDNA haplotype	Genbank accession numbers		
			CytB	***ND2***	***NCX***
1	CN02	Hst42	OR353778	OR353912	OR354046
2	ML02	Hst42	OR353825	OR353959	OR354093
2	ML03	Hst40	OR353826	OR353960	OR354094
2	ML04	Hst42	OR353827	OR353961	OR354095
3	CM01	Hst36	OR353780	OR353914	OR354048
3	CM02	Hst42	OR353785	OR353919	OR354053
3	CM03	Hst42	OR353786	OR353920	OR354054
3	CM04	Hst44	OR353787	OR353921	OR354055
3	CM05	Hst41	OR353776	OR353910	OR354044
3	CM06	Hst42	OR353837	OR353971	OR354105
3	CM07	Hst42	OR353840	OR353974	OR354108
4	CO01	Hst42	OR353779	OR353913	OR354047
5	VM01	Hst42	OR353828	OR353962	OR354096
5	VM02	Hst42	OR353832	OR353966	OR354100
5	VM03	Hst34	OR353795	OR353929	OR354063
5	VM04	Hst42	OR353798	OR353932	OR354066
5	VM05	Hst42	OR353799	OR353933	OR354067
5	VM06	Hst42	OR353801	OR353935	OR354069
5	VM07	Hst43	OR353806	OR353940	OR354074
6	FU01	Hst42	OR353824	OR353958	OR354092
6	FU02	Hst42	OR353830	OR353964	OR354098
6	FU03	Hst42	OR353831	OR353965	OR354099
7	VE01	Hst42	OR353833	OR353967	OR354101
7	VE02	Hst38	OR353847	OR353981	OR354115
7	VE03	Hst37	OR353850	OR353984	OR354118
7	VE04	Hst42	OR353852	OR353986	OR354120
7	VE05	Hst38	OR353853	OR353987	OR354121
7	VE06	Hst38	OR353854	OR353988	OR354122
7	VE07	Hst38	OR353855	OR353989	OR354123
7	VE08	Hst38	OR353857	OR353991	OR354125
7	VE10	Hst42	OR353860	OR353994	OR354128
7	VE11	Hst38	OR353861	OR353995	OR354129
7	VE12	Hst42	OR353862	OR353996	OR354130
7	VE13	Hst38	OR353867	OR354001	OR354135
8	SE01	Hit9	OR353811	OR353945	OR354079
8	SE02	Hit9	OR353812	OR353946	OR354080
8	SE03	Hit9	OR353813	OR353947	OR354081
8	SE04	Hit9	OR353814	OR353948	OR354082
8	SE05	Hit9	OR353755	OR353889	OR354023
8	SE06	Hst39	OR353783	OR353917	OR354051
8	SE07	Hit9	OR353846	OR353980	OR354114

Site code	Specimen	MtDNA haplotype	Genbank accession numbers		
			CytB	ND2	NCX
8	SE08	Hit9	OR353849	OR353983	OR354117
9	CS01	Hit11	OR353809	OR353943	OR354077
9	CS02	Hit9	OR353810	OR353944	OR354078
9	CS03	Hit9	OR353815	OR353949	OR354083
9	CS04	Hit9	OR353816	OR353950	OR354084
9	CS05	Hit9	OR353817	OR353951	OR354085
9	CS06	Hit9	OR353818	OR353952	OR354086
9	CS07	Hit9	OR353819	OR353953	OR354087
9	CS08	Hit10	OR353820	OR353954	OR354088
9	CS09	Hit10	OR353821	OR353955	OR354089
9	CS10	Hit9	OR353822	OR353956	OR354090
9	CS11	Hit6	OR353781	OR353915	OR354049
9	CS12	Hit9	OR353782	OR353916	OR354050
9	CS13	Hit9	OR353784	OR353918	OR354052
9	CS14	Hit9	OR353764	OR353898	OR354032
9	CS15	Hit9	OR353765	OR353899	OR354033
10	GA01	Hab5	OR353834	OR353968	OR354102
10	GA02	Hab5	OR353835	OR353969	OR354103
10	GA03	Hab5	OR353839	OR353973	OR354107
10	GA04	Hab5	OR353841	OR353975	OR354109
10	GA05	Hab5	OR353842	OR353976	OR354110
10	GA06	Hab5	OR353843	OR353977	OR354111
11	RE01	Hit9	OR353829	OR353963	OR354097
11	RE02	Hit9	OR353793	OR353927	OR354061
11	RE03	Hit9	OR353796	OR353930	OR354064
12	CP01	Hit5	OR353790	OR353924	OR354058
12	CP02	Hit9	OR353791	OR353925	OR354059
12	CP03	Hit7	OR353803	OR353937	OR354071
12	CP04	Hit9	OR353804	OR353938	OR354072
12	CP05	Hit9	OR353807	OR353941	OR354075
12	CP06	Hit9	OR353808	OR353942	OR354076
12	CP07	Hit9	OR353760	OR353894	OR354028
13	RG01	Hst42	OR353792	OR353926	OR354060
13	RG02	Hst42	OR353794	OR353928	OR354062
13	RG03	Hst42	OR353762	OR353896	OR354030
14	CG01	Hit6	OR353758	OR353892	OR354026
14	CG02	Hit9	OR353756	OR353890	OR354024
14	CG03	Hit12	OR353797	OR353931	OR354065
14	CG04	Hit9	OR353800	OR353934	OR354068
14	CG05	Hit9	OR353802	OR353936	OR354070
15	DS01	Hit8	OR353759	OR353893	OR354027
16	TG01	Hab11	OR353805	OR353939	OR354073
16	TG02	Hab5	OR353836	OR353970	OR354104
16	TG03	Hab10	OR353838	OR353972	OR354106
16	TG04	Hab9	OR353844	OR353978	OR354112

Site code	Specimen	MtDNA haplotype	Genbank accession numbers		
			CytB	ND2	NCX
17	ST01	Hab1	OR353823	OR353957	OR354091
17	ST02	Hab1	OR353788	OR353922	OR354056
17	ST03	Hab8	OR353848	OR353982	OR354116
17	ST04	Hab1	OR353851	OR353985	OR354119
17	ST05	Hab1	OR353856	OR353990	OR354124
17	ST06	Hab1	OR353858	OR353992	OR354126
17	ST07	Hab1	OR353863	OR353997	OR354131
17	ST08	Hab1	OR353864	OR353998	OR354132
17	ST09	Hab8	OR353866	OR354000	OR354134
18	BI01	Hab6	OR353768	OR353902	OR354036
18	BI02	Hab7	OR353774	OR353908	OR354042
18	BI03	Hab6	OR353845	OR353979	OR354113
18	BI04	Hab6	OR353859	OR353993	OR354127
18	BI05	Hab6	OR353865	OR353999	OR354133
19	PS01	Hst45	OR353766	OR353900	OR354034
19	PS02	Hst35	OR353767	OR353901	OR354035
19	PS03	Hst38	OR353769	OR353903	OR354037
19	PS04	Hst35	OR353770	OR353904	OR354038
19	PS05	Hst35	OR353771	OR353905	OR354039
19	PS06	Hst35	OR353772	OR353906	OR354040
19	PS07	Hst35	OR353773	OR353907	OR354041
19	PS08	Hst35	OR353775	OR353909	OR354043
19	PS09	Hst35	OR353777	OR353911	OR354045
19	PS10	Hst35	OR353868	OR354002	OR354136
19	PS11	Hst35	OR353869	OR354003	OR354137
19	PS12	Hst35	OR353870	OR354004	OR354138
19	PS13	Hst35	OR353871	OR354005	OR354139
19	PS14	Hst35	OR353872	OR354006	OR354140
19	PS15	Hst38	OR353873	OR354007	OR354141
19	PS16	Hst35	OR353874	OR354008	OR354142
19	PS17	Hst38	OR353875	OR354009	OR354143
20	LL01	Hst42	OR353761	OR353895	OR354029
20	LL02	Hst42	OR353763	OR353897	OR354031
20	LL03	Hst42	OR353754	OR353888	OR354022
20	LL04	Hst42	OR353757	OR353891	OR354025
21	LI01	Hit4	OR353789	OR353923	OR354057
*	ABE1	Hab1	OR353876	OR354010	OR354144
*	APU1	Hab1	OR353885	OR354019	OR354153
*	ABE12	Hab2	OR353878	OR354012	OR354146
*	ABE10	Hab2	OR353877	OR354011	OR354145
*	AFO9	Hab3	OR353881	OR354015	OR354149
*	AFO2	Hab4	OR353879	OR354013	OR354147
*	AFO3	Hab5	OR353880	OR354014	OR354148

* new reference individuals for *Speleomantes ambrosii bianchi*
